# Novel Cerium- and Terbium-Doped Gadolinium Fluoride Nanoparticles as Radiosensitizers with Pronounced Radiocatalytic Activity

**DOI:** 10.3390/biomedicines13071537

**Published:** 2025-06-24

**Authors:** Nikita A. Pivovarov, Danil D. Kolmanovich, Nikita N. Chukavin, Irina V. Savintseva, Nelli R. Popova, Alexander E. Shemyakov, Arina D. Filippova, Maria A. Teplonogova, Alexandra V. Yurkovskaya, Ivan. V. Zhukov, Azamat Y. Akkizov, Anton L. Popov

**Affiliations:** 1 Institute of Theoretical and Experimental Biophysics of the Russian Academy of Sciences, Pushchino 142290, Russia; nikitapivovarov.workmail@gmail.com (N.A.P.); kdd100996@mail.ru (D.D.K.); chukavinnik@gmail.com (N.N.C.); savintseva_irina@mail.ru (I.V.S.); nellipopovaran@gmail.com (N.R.P.); alshemyakov@yandex.ru (A.E.S.); 2 Physical Technical Center, Lebedev Physical Institute, Russian Academy of Sciences, Protvino 142281, Russia; 3 Kurnakov Institute of General and Inorganic Chemistry of the Russian Academy of Sciences, Moscow 119991, Russia; arifilippova@yandex.ru (A.D.F.); m.teplonogova@gmail.com (M.A.T.); 4 International Tomography Center, Siberian Branch of the Russian Academy of Sciences, 3A Institutskaya St., Novosibirsk 630090, Russia; yurk@tomo.nsc.ru (A.V.Y.); i.zhukov@tomo.nsc.ru (I.V.Z.); 5 Institute of Mathematics and Natural Sciences, Kabardino-Balkarian State University named after H. M. Berbekov, Nalchik 360004, Russia; akkizov@mail.ru; 6 Scientific and Educational Center, Federal State University of Education, Moscow 107996, Russia

**Keywords:** nanoparticles, radiosensitizer, radiocatalytic activity, cerium, gadolinium, fluoride, X-ray irradiation, proton beam irradiation

## Abstract

**Background:** The use of nanoradiosensitizers is a promising strategy for the precision enhancement of tumor tissue damage during radiotherapy. **Methods:** Here, we propose a novel biocompatible theranostic agent based on gadolinium fluoride doped with cerium and terbium (Gd_0.7_Ce_0.2_Tb_0.1_F_3_ NPs), which showed pronounced radiocatalytic activity when exposed to photon or proton beam irradiation, as well as remarkable MRI contrast ability. A scheme for the production of biocompatible colloidally stable Gd_0.7_Ce_0.2_Tb_0.1_F_3_ NPs was developed. Comprehensive physicochemical characterization of these NPs was carried out, including TEM, SEM, XRD, DLS, and EDX analyses, as well as UV–vis spectroscopy and MRI relaxation assays. **Results:** Cytotoxicity analysis of Gd_0.7_Ce_0.2_Tb_0.1_F_3_ NPs in vitro and in vivo revealed a high level of biocompatibility. It was shown that Gd_0.7_Ce_0.2_Tb_0.1_F_3_ NPs effectively accumulate in MCF-7 tumor cells. A study of their radiosensitizing activity demonstrated that the combined effect of Gd_0.7_Ce_0.2_Tb_0.1_F_3_ NPs and X-ray irradiation leads to a dose-dependent decrease in mitochondrial membrane potential, a sharp increase in the level of intracellular ROS, and the subsequent development of radiation-induced apoptosis. **Conclusions:** This outstanding radiosensitizing effect is explained by the radiocatalytic generation of reactive oxygen species by the nanoparticles, which goes beyond direct physical dose enhancement. It emphasizes the importance of evaluating the molecular mechanisms underlying the sensitizing effectiveness of potential nanoradiosensitizers before choosing conditions for their testing in in vivo models.

## 1. Introduction

Radiation therapy plays an important role in cancer treatment [1,2,3]. Technical advances in the delivery of a dose of high-energy photons or protons have contributed to more consistent tumor irradiation with decreased damage to normal tissues [4,5]. However, cancer radiation resistance and side effects remain major challenges for successful tumor radiotherapy; hence, additional approaches are needed to improve treatment effectiveness [6,7,8]. The use of functional nanomaterial-based radiosensitizers represents a promising strategy for improving the tissue specificity of radiation therapy [9,10,11,12,13,14,15]. To date, the main concept in the creation of novel radiosensitizers is the use of high-Z elements, which is based on a higher probability of interaction of atoms of such elements with ionizing radiation and a strong local increase in the absorbed dose [16,17,18]. For example, hafnium oxide nanoparticles (NBTRX3) have already been approved in the EU for clinical use in the treatment of locally advanced soft tissue sarcoma [19,20,21] by intratumoral administration. Additionally, gadolinium-containing nanoparticles (AGuIX) are in the 3rd stage of clinical trials for therapy in patients with multiple brain metastases through intravenous injection [22,23,24]. Thus, the strategy of using high-Z elements as the basis of nanoradiosensitizers is gaining wide application in clinical practice. Meanwhile, recent works show that the use of high-Z elements is not always effective. A key success factor in using nanoradiosensitizers may be their radiocatalytic activity, which promotes the formation of reactive oxygen species (ROS). This determines the success and effectiveness of radiosensitization of cancer cells [25,26,27]. At this point, semiconductor nanoparticles that promote the formation of ROS under irradiation by forming electron-hole pairs or nanoparticles based on transition metals are promising candidates for radiotherapy [28,29].

In particular, cerium-containing nanoparticles (nanoceria) are known for their remarkable pH-dependent redox activity and are considered a promising nanomaterial for the development of selective radiosensitizers and radioprotectors [30,31]. Ceria can exist in two valence states: partially reduced trivalent cerium Ce^3+^ and fully oxidized tetravalent cerium Ce^4+^ [32]. The presence of cerium atoms in the crystal lattice of nanoparticles in these two states causes reduction–oxidation reactions on their surface. Cerium dioxide nanoparticles (CeO_2_ NPs) also belong to the class of inorganic nanozymes capable of mimicking the catalytic activity of natural enzymes such as superoxide dismutase (SOD), catalase (CAT), phosphatase, and peroxidase, using their unique ability of auto-regeneration [33,34,35,36,37]. The catalytic activity of CeO_2_ NPs is determined by the nonstoichiometry of Ce^3+^/Ce^4+^ and oxygen atoms on their surface and strongly depends on the synthesis methods and stabilizers used [38,39,40,41]. The ultra-sensitivity of the cerium oxide surface to the composition of the microenvironment makes it possible to adjust its electronic configuration and change the direction of bioactivity from antioxidant to prooxidant [42]. Zhang L. et al. previously shown that cerium-containing nanoparticles exhibit selective redox activity under UV irradiation [43]. Pyrroloquinoline quinone-modified CeO_2_ NPs demonstrated radiation-induced selective cytotoxicity toward normal NCTC L929 cells and EMT6/P cancer cells [44]. Doping CeO_2_ NPs with the ions of transition and rare earth elements (REEs) endows them with new functional features, including enhanced oxygen nonstoichiometry [45,46,47] and MRI imaging [48,49,50]. Previously, we showed that cerium fluoride nanoparticles exhibit redox activity, selectively modulate cellular responses under X-ray irradiation [51], and have antiviral effects against vesicular stomatitis virus infection [52]. Moreover, cerium fluoride nanoparticles can be used in X-ray-induced photodynamic therapy (X-PDT). Kornienko A. et al. proposed a novel type of nanoscintillator based on cerium fluoride nanoparticles modified with flavin mononucleotide. This nanoscintillator demonstrated selective cytotoxicity to tumor epidermoid carcinoma cells under X-PDT, increasing the radiosensitivity of cancer cells by 1.9 times [53]. Thus, the combination of both high-Z elements and catalytically active centers in the nanoradiosensitizer should provide a synergistic effect of radiosensitizing action against tumor cells.

In this study, we developed a scheme for producing colloidally stable cerium- and terbium-doped gadolinium fluoride nanoparticles (Gd_0.7_Ce_0.2_Tb_0.1_F_3_ NPs) and conducted a comprehensive characterization of their physicochemical properties and biocompatibility in vitro and in vivo. Furthermore, we performed a comparative assessment of the radiocatalytic activity of Gd_0.7_Ce_0.2_Tb_0.1_F_3_ NPs under photon and proton beam irradiation. After in-depth characterization of the Gd_0.7_Ce_0.2_Tb_0.1_F_3_ NPs and a quantitative assessment of ROS generation under cell-free conditions, we provided information on the molecular mechanisms of the radiosensitizing effect of Gd_0.7_Ce_0.2_Tb_0.1_F_3_ NPs in thee MCF-7 breast carcinoma cell line. An integrated approach to studying the bioactivity of novel nanoradiosensitizers must necessarily include an analysis of their molecular mechanisms of action under irradiation conditions. This makes it possible to validate and optimize the conditions for their use, as well as to determine the most effective parameters for binary cancer radiotherapy technologies, including irradiation type (photons or proton beam), energy, tumor type, etc.

## 2. Materials and Methods

### 2.1. Synthesis of Gd_0.7_Ce_0.2_Tb_0.1_F_3_ NPs

The initial substances for the creation of Gd_0.7_Ce_0.2_Tb_0.1_F_3_ nanoparticles were GdCl_3_·6H_2_O (Lanhit, Moscow, Russia, 99.99%), CeCl_3_·7H_2_O (Lanhit, Russia, 99.9%), TbCl_3_·6H_2_O (Lanhit, Russia, 99.99%), hydrofluoric acid (40% aqueous solution, Sigma Tech), ammonium citrate (Sigma Aldrich, >98%), and isopropanol (Himmed, Moscow, Russia). The rare earth element chlorides (1.3 g of GdCl_3_·6H_2_O, 0.373 g of CeCl_3_·7H_2_O, and 0.187 g of TbCl_3_·6H_2_O) were dissolved in 15 mL of distilled water while stirring on a magnetic stirrer. Then, 150 mL of isopropanol was added to the resulting solution. Subsequently, 0.885 mL of 40% hydrofluoric acid (HF) was dissolved in 50 mL of isopropanol. The resulting solution was added to the solution of rare earth element chlorides, and the mixture was stirred vigorously. The resulting precipitate was separated by filtering through a paper filter and rinsed three times with isopropanol. The precipitate was then placed in a drying cabinet for 30 min at 50 °C to remove the isopropanol. The resulting wet, gel-like precipitate was then redispersed in 100 mL of deionized water. A solution of ammonium citrate was added to the resulting suspension, with a ratio of nanoparticles to citrate of 1:1. This resulted in the formation of a concentrated sol.

### 2.2. Characterization of Gd_0.7_Ce_0.2_Tb_0.1_F_3_ NPs

Gd_0.7_Ce_0.2_Tb_0.1_F_3_ NPs were investigated using a Tescan Amber GMH (Tescan Group, Brno-Kohoutovice, Czech Republic) scanning electron microscope (SEM) at an accelerating voltage of 30 kV, using an R-STEM detector. SEM images were captured using an Everhart–Thornley detector at a magnification of 75,000 to 225,000 and an accelerating voltage of 1 kV. The elemental composition of the samples was determined using energy dispersive X-ray spectroscopy (EDX) on a Tescan Amber GMH microscope, equipped with an Ultim MAX detector with a 100 mm^2^ active area (Oxford Instruments, Abingdon, UK), at an accelerating voltage of 20 kV. A drop of the aqueous solution was placed on a formvar/carbon Cu grid (Ted Pella Inc., Redding, CA, USA) and allowed to dry. The images were captured in dark field mode at a magnification of 300,000. The size and shape of the Gd_0.7_Ce_0.2_Tb_0.1_F_3_ nanoparticles were analyzed using transmission electron microscopy (TEM) on a Leo912 AB Omega electron microscope (Zeiss, Oberkochen, Germany). The samples were placed on copper grids with a diameter of 3.05 mm and a polymer film coating. The samples were subjected to X-ray diffraction analysis using a DX-2700BH diffractometer (Haoyuan, Dandong, China). The analysis was conducted using CuKα radiation with a wavelength of 1.54184 Å. The diffractometer scanned the samples in the range of 5–60 degrees in 2θ, with a step size of 0.02 degrees and a shutter speed of 1 s per step. The diffractograms were then compared to the ICDD PDF2 database for indexing.

The concentration of the Gd_0.7_Ce_0.2_Tb_0.1_F_3_ nanoparticles in solution was determined using a gravimetric method. The corundum crucibles, which had been pre-heated at 900 °C for two hours, were weighed using analytical scales to determine their initial weight. Then, three milliliters of the solution were placed in each crucible, and the crucibles were heated in a muffle furnace at 900 °C for two hours, with a slow heating rate of approximately 3° per minute. After cooling to room temperature, the crucibles were weighed again. The weight of the dry residue from the annealing process was determined, and the concentration of the initial solution was calculated.

The absorbance of the Gd_0.7_Ce_0.2_Tb_0.1_F_3_ nanoparticles in solution was measured using a DS 11+ spectrophotometer (DeNovix, Wilmington, DE, USA) in the ultraviolet–visible range. The measurements were conducted in the wavelength range from 200 to 600 nm, with increments of 0.1 nm. The distribution of particle sizes and their ζ-potentials were measured in water (18.2 mω·cm), cultural medium DMEM/F12, and DMEM/F12 + 10% FBS using dynamic and electrophoretic light scattering techniques at 25 °C. The measurements were conducted using a BeNano 90 Zeta analyzer (BetterSize, Dandong, China). The analyzer employed a 90° detection angle and a 671 nm solid-state laser with a power of 50 MW.

The stability of the synthesized Gd_0.7_Ce_0.2_Tb_0.1_F_3_ NPs colloidal solution was assessed over a period of 28 days. The size and zeta potential of the nanoparticles were measured every 7 days, as shown in Table S1. The fluorescence spectrum of the nanoparticles was obtained using a Cary Eclipse spectrofluorimeter. The measurements were performed in a quartz cuvette with transparent walls. The delay time was set to 0.1 ms, and the excitation and emission slits were both set to 5 nm. The longitudinal relaxation rate was measured using a 200 MHz NMR spectrometer with a magnetic field of 4.7 T. No deuterated water was added, and the NMR measurements were taken without field stabilization using the lock signal. The temperature of the sample during the measurements was 25 °C. A standard t1ir relaxation–inversion-recovery pulse sequence was used, with a relaxation delay of d1 = 15 s, 16 delay values for restoring magnetization after a 180° pulse from 20 ms to 15 s, and a number of scans of NS = 2. The obtained relationships between the intensity of the NMR signal of water and the time elapsed after the 180° pulse were fitted using a single exponential function. Based on this fit, the time of longitudinal relaxation of protons, T1, was determined, and the total relaxation rate R1 (total) = 1/T1 was calculated. The relaxation rate of protons in pure water used for dilution was also measured: R1 (H_2_O) = 1/T1 (H_2_O) = 0.31 s^−1^. The change in the relaxation rate of protons due to the presence of paramagnetic nanoparticles was calculated as R1 = R1 (total) − R1 (H_2_O). The relaxivity value, r1, of the sample was determined by fitting the dependence of R1 on [Gd]. The full scheme of experiments with Gd_0.7_Ce_0.2_Tb_0.1_F_3_ NPs is shown in Figure S1.

### 2.3. Cytotoxicity Analysis

#### 2.3.1. Cell Lines

The experiments were conducted using three different types of cell lines: B16/F10 (a murine melanoma cancer cell line), MCF-7 (a human adenocarcinoma cancer cell line), and NCTC L929 (a normal murine fibroblast cell line), which were obtained from the cryostorage of the Theranostics and Nuclear Medicine Laboratory at the Institute of Theoretical and Experimental Biophysics of the Russian Academy of Sciences in Pushchino, Russia. The cells were grown in a culture medium consisting of DMEM/F12 (1:1) (PanEko, Moscow, Russia) supplemented with 50 µg/mL of penicillin (PanEko, Russia), 50 µg/mL of streptomycin (PanEko, Russia), 10% fetal bovine serum (FBS) (HyClone, Logan, UT, USA), and 146 mg of L-glutamine (PanEko, Russia). Cells were cultured at 37 °C in an atmosphere with 95% humidity, consisting of 95% air and 5% CO_2_.

#### 2.3.2. MTT Assay

Cells were placed in 96-well plates at a density of 2.5 × 10^4^ cells per cm^2^. The Gd_0.7_Ce_0.2_Tb_0.1_F_3_ nanoparticles were allowed to interact with the cells for 72 h. After 72 h of this interaction, MTT solution (3-(4,5-dimethylthiazol-2-yl)-2,5-diphenyltetrazolium bromide, PanEko, Moscow, Russia) (0.5 mg/mL) was added to the wells, along with the culture medium without fetal bovine serum. The mixture was then incubated in an incubator for three hours. Afterward, the culture medium was removed, and the formazan crystals were dissolved using DMSO. The optical density of the resulting solutions was then measured at a wavelength of 570 nm using an INNO-S plate reader (LTEK, Seongnam-si, Republic of Korea). The cells were exposed to Gd_0.7_Ce_0.2_Tb_0.1_F_3_ NPs at concentrations ranging from 0.1 to 1 mM. The control group cells were not exposed to Gd_0.7_Ce_0.2_Tb_0.1_F_3_ NPs.

#### 2.3.3. Live/Dead Assay

The cytotoxic impact of Gd_0.7_Ce_0.2_Tb_0.1_F_3_ nanoparticles was evaluated using a Live/Dead assay. This method involves measuring the proportion of dead cells relative to the total number of cells after exposure to Gd_0.7_Ce_0.2_Tb_0.1_F_3_ nanoparticles. The cells were labeled with Hoechst 33342 (a dye that stains the nuclei of all cells, at a concentration of 5 µM) and propidium iodide (a dye that stains the nuclei of dead cells, at a concentration of 5 µM) after 72 h of exposure. The cells were then photographed using a ZOE fluorescent imager (Bio-Rad, Hercules, CA, USA). The number of cells was counted using ImageJ software (1.54p version). Three different areas of the field were analyzed in three different microphotographs. The results of the quantitative analysis were presented as mean ± standard deviation.

#### 2.3.4. Mitochondrial Membrane Potential (MMP) Analysis

MMP was assessed using TMRE (tetramethylrhodamine, ethyl ester) fluorescent dye from ThermoFisher, Carlsbad, CA, USA. After 72-h incubation with Gd_0.7_Ce_0.2_Tb_0.1_F_3_ NPs, the culture medium was replaced with a TMRE solution (1µM) in HBSS (Hank’s Balanced Salt Solution, PanEko, Moscow, Russia) for 15 min. Cells were then washed three times with HBSS and photographed using a ZOE fluorescent imager (Bio-Rad, Hercules, CA, USA). TMRE fluorescence intensity, which directly correlates with MMP, was quantified using ImageJ software. Three distinct regions on each micrograph were analyzed, and the results were presented as mean ± standard deviation. MMP of irradiated cells was studied one hour after irradiation by flow cytometry. To do this, after irradiation, cells were washed 3 times, trypsin-EDTA was added, and the cells were then transferred to a thermostat (37 °C) until they were detached. The cell suspension was then centrifuged at 2000 rpm for 2 min. After removal of the supernatant, the cellular precipitate was resuspended in a PBS buffer without Ca^2+^ and Mg^2+^, followed by the addition of TMRE (Lumiprobe, Russia) at a concentration of 5 µM. The cells were mixed using a vortex and then redispersed using a pipette. Samples were then taken and analyzed using a BeamCyte-1026M flow cytometer.

#### 2.3.5. Detection of Free Gadolinium Ions

The leaching of Gd^3+^ ions from Gd_0.7_Ce_0.2_Tb_0.1_F_3_ NPs was evaluated using absorption spectrometry with an arsenazo III indicator. For this purpose, a calibration curve was constructed for saline solutions containing fixed concentrations of gadolinium (from 1 µm to 1000 µm) using Gd (NO_3_)_3_ * 6H_2_O as a water-soluble gadolinium salt. Arsenazo III solution (10 µM) was prepared in 0.1 M acetate buffer at pH 5.0, and aliquots of 10 µL of Gd (NO_3_) solutions at different concentrations were placed in wells. Gd_0.7_Ce_0.2_Tb_0.1_F_3_ NPs were mixed separately in a saline solution (0.9 wt.% NaCl) for 24 h at 23 °C, followed by dialysis using a membrane with a cut-off of 1. Then, 10 µL of the dialysis supernatant was added to the wells containing arsenazo III. The liquids in the wells were thoroughly mixed at 500 RPM for 15 min, and the optical density of the solutions was measured using an INNO-S microplate reader (LTEK, Korea) at 655 nm.

### 2.4. Irradiation Set-Up

#### 2.4.1. Photon Irradiation

X-ray treatment was performed using the X-ray therapeutic device RTM-13, manufactured by Mosrentgen in Moscow, Russia. The suspension of Gd_0.7_Ce_0.2_Tb_0.1_F_3_ nanoparticles was exposed to X-ray radiation at doses ranging from 0 to 5 Gy, with a dose rate of 1 Gy per minute. The X-ray machine was set to operate at a voltage of 200 kV, a focal length of 37.5 cm, and a current of 20 mA. The suspension was placed in 2 mL plastic tubes. The uniformity of the dose and the size of the field were monitored using an EBT3 radiometric film.

#### 2.4.2. Proton Beam Irradiation

The “Prometeus” proton therapy system (PROTOM, Protvino, Russia) was used to irradiate cells. The energy selected for irradiation in the “on-flight” mode was 150 MeV. The uniformity and size of the field were monitored using an EBT3 radiometric film, while the absorbed dose was monitored using a PTW Unidos webline electrometer with a PTW PinPoint 3D 31022 ionization chamber. Additionally, a PTW Bragg Peak 34073 ionization chamber was used for dosimetry at the Bragg Peak. The uniformity at the 95% isodose level was 98%. The Gd_0.7_Ce_0.2_Tb_0.1_F_3_ NPs suspension was placed in 2 mL plastic tubes.

### 2.5. Acellular ROS Detection Assays

#### 2.5.1. H2DCFDA Assay

H2DCFDA was used to determine the level of ROS in cells, as it is oxidized by ROS to produce the fluorescent compound dichlorofluorescein (DCF). To remove the acetyl group from this compound, the dye solution was incubated with 0.01 M NaOH in the dark for 30 min at 0 °C to allow the reaction to occur. After that, the DCF solution was mixed with 15 mL of Tris-HCl buffer (0.005 M, pH 6.5, 7.4) to prepare the samples for analysis. To measure ROS levels in the presence of Gd_0.7_Ce_0.2_Tb_0.1_F_3_ NPs, the samples were divided into four groups:

1. DCF solution;

2. DCF with Gd_0.7_Ce_0.2_Tb_0.1_F_3_ NPs;

3. DCF under irradiation;

4. DCF + Gd_0.7_Ce_0.2_Tb_0.1_F_3_ NPs + irradiation.

The final concentrations of the NPs in groups 2 and 4 were 0.01, 0.05, and 0.1 mM, respectively. The fluorescence of the samples exposed to radiation was measured using a Biotek Synergy H1 spectrofluorometer at a wavelength of 488 nm for excitation and 525 nm for emission. To assess the level of reactive oxygen species (ROS) under irradiation in the presence of Gd_0.7_Ce_0.2_Tb_0.1_F_3_ NPs, the experimental samples were divided into four groups: (i) a solution of DCF, (ii) a solution of DCF with Gd_0.7_Ce_0.2_Tb_0.1_F_3_ NPs alone, (iii) a solution of DCF exposed to radiation alone, and (iv) a solution of DCF with Gd_0.7_Ce_0.2_Tb_0.1_F_3_ NPs and exposed to radiation. The final concentrations of Gd_0.7_Ce_0.2_Tb_0.1_F_3_ NPs in groups (ii) and (iv) were 0.01, 0.05, and 0.1 mM. The fluorescence of the irradiated solutions was then measured using a Biotek Synergy H1 spectrofluorometer (λex = 488 nm; λem = 525 nm). The efficiency of ROS generation was estimated as *DEF_ROS_* by the following formula:

(1) $${DEF}_{ROS}\;=\;\frac{{FI}_{n\;Gy\;with\;NPs}\;-\;{FI}_{0\;Gy\;with\;NPs}}{{FI}_{n\;Gy\;without\;NPs}\;-{\;FI}_{0\;Gy\;without\;NPs}}$$

where *FI*_(*x*)_ is the average value of absolute fluorescence indices in the group.

#### 2.5.2. NBT Assay

The formation of superoxide anion ^•^O_2_^−^ in a suspension of Gd_0.7_Ce_0.2_Tb_0.1_F_3_ NPs under radiation was investigated by measuring the change in the optical density of blue nitrotetrazolium chloride (NBT) at a wavelength of 260 nm. Aqueous solutions of 1 mL Gd_0.7_Ce_0.2_Tb_0.1_F_3_ nanoparticles with a concentration of 0.1 mM and 1 mL NBT with a concentration of 30 µg/mL were combined in a 2 mL centrifuge tube. The solution was then subjected to ultrasonic dispersion and exposed to X-rays for 4 min at a dose of 8 Gy. Afterward, the solutions were centrifuged at 12,000 rpm for 5 min. The resulting supernatants were analyzed using a DS-11 spectrophotometer (DeNovix, Wilmington, DE, USA) to measure their optical density at 260 nm.

#### 2.5.3. TA Assay

The formation of hydroxyl radical (^•^OH) due to X-ray radiation in a suspension of Gd_0.7_Ce_0.2_Tb_0.1_F_3_ NPs was measured using disodium terephthalate (TA). The experiment involved six groups: a control group; a group with TA; a group with Gd_0.7_Ce_0.2_Tb_0.1_F_3_ NPs; a group with TA and Gd_0.7_Ce_0.2_Tb_0.1_F_3_ NPs; a group with TA and irradiation; and a group with TA, Gd_0.7_Ce_0.2_Tb_0.1_F_3_ NPs, and irradiation. Solutions containing TA (0.3 mL, 5 mmol/L) and Gd_0.7_Ce_0.2_Tb_0.1_F_3_ NPs (0.3 mL, 0.5 mmol/L) were added to phosphate-buffered saline (PBS) (1.5 mL, 0.2 mol/L, pH 6.5 and 7.4) and then diluted with deionized water to a total volume of 3 mL. Some of the groups were exposed to X-ray photons at a dose of 8 Gy for 4 min. The experimental tubes were then incubated overnight at 37 °C in the dark. The fluorescence signal was measured at a wavelength of 435 nm when excited with a wavelength of 315 nm using a Cary Eclipse cuvette spectrofluorimeter (Agilent, Santa Clara, CA, USA).

### 2.6. Flow Cytometry

#### 2.6.1. Intracellular Reactive Oxygen Species (ROS) Level Analysis

The investigation of intracellular reactive oxygen species was conducted one hour after irradiation using flow cytometry. The cells were collected and washed three times with a phosphate-buffered saline (PBS) solution without calcium and magnesium ions, and then detached from the plate by adding trypsin-EDTA. The cell suspension was centrifuged at 2000 revolutions per minute for two minutes. After removing the supernatant, the cell pellet was resuspended in a PBS solution without calcium and magnesium ions, and then 2,7-H2DCFDA (2′,7′-dichlorofluorescein diacetate) fluorescent dye (Lumiprobe, Russia) was added at a concentration of 5 micromolar. The cells were mixed using a vortex and then resuspended using a pipette. Samples were then taken and analyzed using a BeamCyte-1026M flow cytometer.

#### 2.6.2. Mitochondrial Membrane Potential (MMP) Analysis

MMP was investigated one hour after exposure to radiation using flow cytometry. To accomplish this, after irradiation, the cells were rinsed three times, and then trypsin-EDTA was added. The cells were then transferred to a thermostat (37 °C) and allowed to detach. The cell suspension was then centrifuged at 2000 rpm for two minutes. After removing the supernatant, the cellular pellet was resuspended in a PBS buffer lacking Ca^2+^ and Mg^2+^. Next, TMRE (Lumiprobe, Russia) was added at a concentration of 5 µM. The cells were mixed using a vortex and then resuspended using a pipette. Samples were then collected and analyzed using a BeamCyte-1026M flow cytometer.

#### 2.6.3. Apoptosis Analysis

After 72 h of X-ray exposure, the cells were collected and rinsed three times with a phosphate-buffered saline solution without calcium and magnesium (PanEko, Russia). The cells were detached from the plate by adding trypsin-EDTA (PanEko, Russia). The cells were then rinsed once more with a cold binding buffer from the apoptosis staining kit (Lumiprobe, Moscow, Russia) and resuspended in the same buffer. To the cell suspensions, we added solutions of annexin V-AF488 (a marker for apoptosis, which binds to phosphatidylserine on the outer surface of the cell membrane and fluoresces in the FITC channel) and propidium iodide (a dye that intercalates into the nucleic acids of dead cells and fluoresces in the PE channel). The cell suspensions were then mixed and incubated for 15 min at room temperature in the dark. After the incubation, without any additional rinsing, the binding buffer was added to each sample. The cells were mixed using a vortex and then resuspended using a pipette. The samples were subsequently collected and subjected to analysis using a BeamCyte-1026M flow cytometer manufactured by BeamDiag in Changzhou, China. The software provided by the flow cytometer generated graphs illustrating the distribution of cells into categories based on their status: living (FITC-/PE-), early apoptotic (FITC+/PE-), late apoptotic (FITC+/PE+)*,* and necrotic (FITC-/PE+).

### 2.7. Clonogenic Assay

Cells were inoculated into 12.5 cm^2^ flasks at a density of 3 × 10^5^ cells per flask and cultured for 10 h. Subsequently, Gd_0.7_Ce_0.2_Tb_0.1_F_3_ nanoparticles were added to the cell culture medium, and the flasks were incubated for 16 h (overnight). Afterward, the cell monolayer was gently rinsed three times with HBSS to remove any unbound Gd_0.7_Ce_0.2_Tb_0.1_F_3_ nanoparticles. The flasks were then filled with serum-free culture medium and exposed to X-ray irradiation. Following irradiation, the cells were seeded into 6-well plates at a concentration of 1000 cells per well in a culture medium containing serum. Cells were cultured at 37 °C in an atmosphere containing 5% CO_2_. The formation of colonies was monitored daily using a CloneSelect Imager plate reader (Molecular Devices, San Jose, CA, USA). After the colonies had formed in the control group (8–9 days), the cells were washed three times with PBS, fixed in a 4% paraformaldehyde solution (Sigma Aldrich, St. Louis, MO, USA), and stained with 0.1% crystal violet (PanEko, Moscow, Russia).

### 2.8. Acute Toxicity Analysis of Gd_0.7_Ce_0.2_Tb_0.1_F_3_ NP In Vivo

Male Kv:SHK mice, aged 10–11 weeks and weighing 33 ± 4 g, were used in the experiment. The animals were housed in a controlled environment with a temperature of 23 ± 2 °C. They had access to commercial rodent feed and fresh water throughout the experiment. At the end of the study, the mice were euthanized by cervical dislocation.

Gd_0.7_Ce_0.2_Tb_0.1_F_3_ nanoparticles were administered intraperitoneally at a dose of 4 mg/mL. The control group received a saline solution. The solutions were administered at a volume of 0.3 mL per mouse.

All procedures involving mice were conducted in accordance with international guidelines for working with laboratory animals and the requirements of the Commission on Biosafety and Bioethics of the ITEB RAS (Minutes of the Commission on Biosafety and Bioethics of the ITEB RAS No. 25/2021, dated 9 February 2021).

The acute toxicity of Gd_0.7_Ce_0.2_Tb_0.1_F_3_ nanoparticles was evaluated in mice using a 20-day survival test. The control group consisted of five animals, while the experimental group consisted of fifteen. Furthermore, blood samples were collected at specific time intervals post-administration: 0, 2, 5, 7, and 14 days. The blood was collected from the caudal vein by cutting off the tip of the tail. Measurements were conducted using a hematological analyzer (model DH 36Vet, manufactured by Dymind in China).

### 2.9. Results Processing

Diagrams were created using GraphPad Prism software (8.0.0 Version). The results are presented as mean ± standard deviation (SD).

## 3. Results

**Figure 1:**
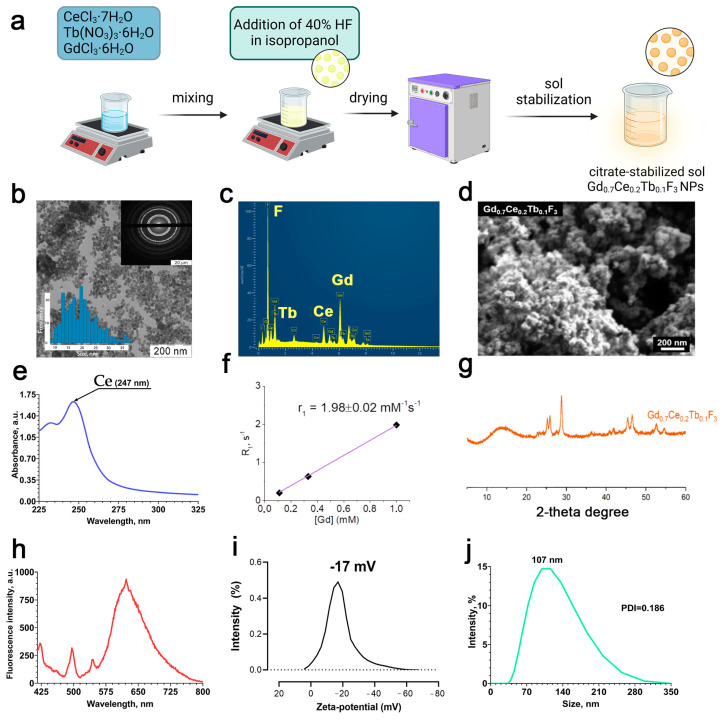
Synthesis scheme (**a**), TEM image and selected area electron diffraction (in the inset) (**b**), EDX spectrum (**c**), SEM image (**d**), UV–visible absorbance spectrum (**e**), relaxation rate R_1_ (**f**), XRD pattern (**g**), fluorescence spectrum (**h**), ζ-potential distribution in deionized water (**i**), and hydrodynamic size distribution in deionized water (**j**) of Gd_0.7_Ce_0.2_Tb_0.1_F_3_ NPs.

The scheme of Gd_0.7_Ce_0.2_Tb_0.1_F_3_ NPs synthesis is shown in Figure 1a. According to transmission and scanning electron microscopy (Figure 1b,d), the Gd_0.7_Ce_0.2_Tb_0.1_F_3_ precipitate consists of a hexagonal shape structure and spherical morphology with a size of < 50 nm. The chemical composition of the nanoparticles was confirmed by EDX analysis (Figure 1c). According to the results of EDX, the element–metal ratio in Gd_0.7_Ce_0.2_Tb_0.1_F_3_ NPs corresponds to the one established during synthesis. The obtained spectrum of Gd_0.7_Ce_0.2_Tb_0.1_F_3_ NPs in the UV–visible region (Figure 1e) shows an absorption band at a wavelength of 247 nm, which corresponds to the electronic 4f^1^–5d^1^ transition of Ce^3+^. The rate of longitudinal relaxation of protons, R_1_, estimated using a 200 MHz NMR spectrometer (magnetic field 4.7 T), showed that the relaxivity value, r_1_, for Gd_0.7_Ce_0.2_Tb_0.1_F_3_ NPs was 1.98 ± 0.02 mM^−1^s^−1^ (Figure 1f). Figure 1g shows a diffractogram of a precipitate obtained during the synthesis of gadolinium fluoride sol doped with cerium and terbium cations, Gd_0.7_Ce_0.2_Tb_0.1_F_3_. According to the results of XRD, the obtained diffraction pattern corresponds to the diffraction pattern of cerium fluoride (CeF_3_, spatial group P63/mcm, card PDF2 No. 8–45), with reflexes noticeably shifted towards large angles of 2θ. According to Wulff–Bragg’s law, a shift towards large angles indicates a decrease in the parameters of the Gd_0.7_Ce_0.2_Tb_0.1_F_3_ unit cell compared to the CeF_3_ unit cell. A full-profile refinement of the parameters of the fluoride unit cell Gd_0.7_Ce_0.2_Tb_0.1_F_3_ in the TOPAS program using the Le Baille method gave the following values: a = 7.043 (1) Å and c = 7.202 (2) Å (Rwp = 4.54). The corresponding parameters of the unit cell CeF_3_ are 7.112 Å and 7.279 Å. The decrease in unit cell parameters during the transition from CeF_3_ to Gd_0.7_Ce_0.2_Tb_0.1_F_3_ is associated with a decrease in the average cation radius in these compounds: 1.034 Å for CeF_3_ and 0.956 Å for Gd_0.7_Ce_0.2_Tb_0.1_F_3_, according to calculations based on cation radii according to Shannon-Pruitt. The fluorescence spectrum shows the transfer of energy from cerium to terbium within the crystal lattice of Gd_0.7_Ce_0.2_Tb_0.1_F_3_ NPs (Figure 1h). The zeta potential of the nanoparticles in deionized water was −17 ± 0.2 mV, hence the Gd_0.7_Ce_0.2_Tb_0.1_F_3_ NPs are stabilized by the negative charge (Figure 1i). The hydrodynamic diameter of the nanoparticles in deionized water was about 120 ± 5.3 nm with a PDI of 0.108 ± 0.01 (Figure 1j). Therefore, Gd_0.7_Ce_0.2_Tb_0.1_F_3_ NPs form a colloidally stable and uniform nanodisperse system in deionized water. We also analyzed the size and zeta-potential of Gd_0.7_Ce_0.2_Tb_0.1_F_3_ NPs in the culture medium. The nanoparticle sizes in culture medium and culture medium with FBS (10%) were 2047.30 nm (PDI: 0.609) and 93.33 nm (PDI: 0.411), respectively (Figure S2). Thus, it can be concluded that plasma proteins provide stabilization of nanoparticles due to opsonization. The zeta potentials of Gd_0.7_Ce_0.2_Tb_0.1_F_3_ NPs in culture medium and culture medium with FBS (10%) were −9.2798 mV and −12.3691 mV, respectively.

**Figure 2:**
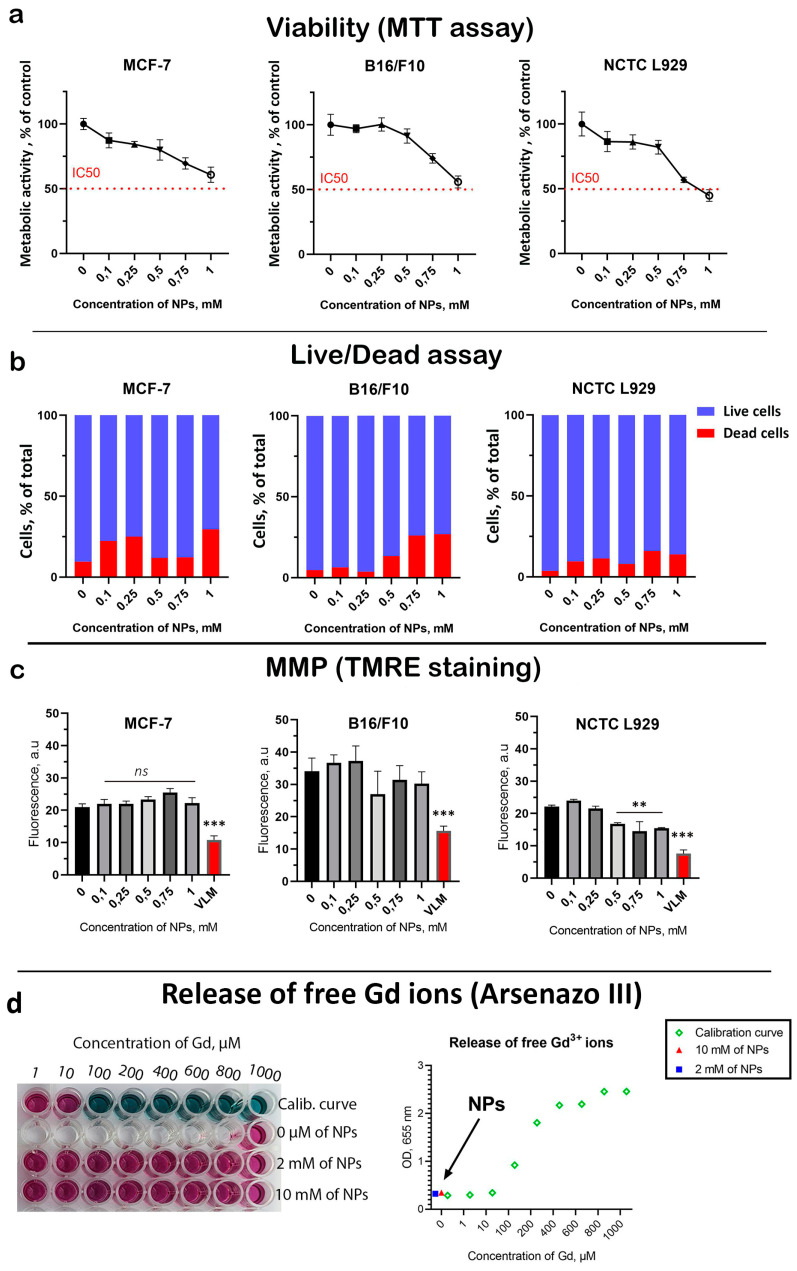
Effect of Gd_0.7_Ce_0.2_Tb_0.1_F_3_ NPs on viability (**a**), death frequency (**b**), and mitochondrial membrane potential (**c**) of MCF-7 (human adenocarcinoma), B16/F10 (mouse melanoma), and NCTC L929 (mouse fibroblasts) cells 72 h after coincubation. Valinomycin (VLM) was used as a positive control for MMP assessment. Results are presented as mean ± SD. The statistical significance of the differences between the control (0 mM) and the experimental groups was confirmed using the Student’s *t*-test with corresponding *p* values: 0.001 < *p* < 0.01 (**), 0.0001 < *p* < 0.001 (***), *ns*- there is no statistical difference with the control. Analysis of free gadolinium content in saline after incubation with Gd_0.7_Ce_0.2_Tb_0.1_F_3_ NPs (**d**).

Next, a comprehensive analysis of the cytotoxicity of Gd_0.7_Ce_0.2_Tb_0.1_F_3_ NPs was performed on three types of cell cultures: B16/F10 (human skin melanoma), MCF-7 (human breast carcinoma), and NCTC L929 (normal mouse fibroblasts). This included the analysis of cell viability (MTT assay) and frequency of cell death (Live/Dead assay) 72 h after coincubation (Figure 2). A dose-dependent decrease in cell viability was revealed for all cell lines (Figure 2a). The highest Gd_0.7_Ce_0.2_Tb_0.1_F_3_ NPs concentration of 1 mM almost halved the viability of both tumor and normal cells. At the same time, NCTC L929 cells turned out to be the most sensitive to the cytotoxic effects of NPs. A dose-dependent increase in the proportion of dead cells was discovered (Figure 2b and Figure S3). However, the frequency of cell death was higher for cancer cells than for normal cells at the same concentrations, indicating a selective cytotoxic effect. Considering that Gd_0.7_Ce_0.2_Tb_0.1_F_3_ NPs have redox activity, we analyzed their effect on mitochondrial metabolism (Figure 2c and Figure S4). A slight (15–20%) dose-dependent decrease in MMP was revealed for all cell lines at the highest Gd_0.7_Ce_0.2_Tb_0.1_F_3_ NPs concentration of 1 mM, which correlates with the cell viability results. One of the possible mechanisms of the toxic effect of gadolinium-containing nanoparticles is the release of free gadolinium ions, which are highly toxic due to the presence of seven unpaired electrons. In this regard, we analyzed free gadolinium ions using a selective indicator—arsenazo III. It was shown that after 24 h of incubation of Gd_0.7_Ce_0.2_Tb_0.1_F_3_ NPs, there is no release of free gadolinium ions from the NPs, which excludes possible serious toxic effects associated with the dissolution of Gd_0.7_Ce_0.2_Tb_0.1_F_3_ NPs. Considering the fact that we used NPs at high concentrations (up to 1 mM), we assume that cytotoxic effects may be associated with the aggregation of Gd_0.7_Ce_0.2_Tb_0.1_F_3_ NPs in the culture medium. Additionally, we did not detect any toxic effects following intraperitoneal administration of Gd_0.7_Ce_0.2_Tb_0.1_F_3_ NPs in mice during a 20-day survival test in vivo (Figure S5).

**Figure 3:**
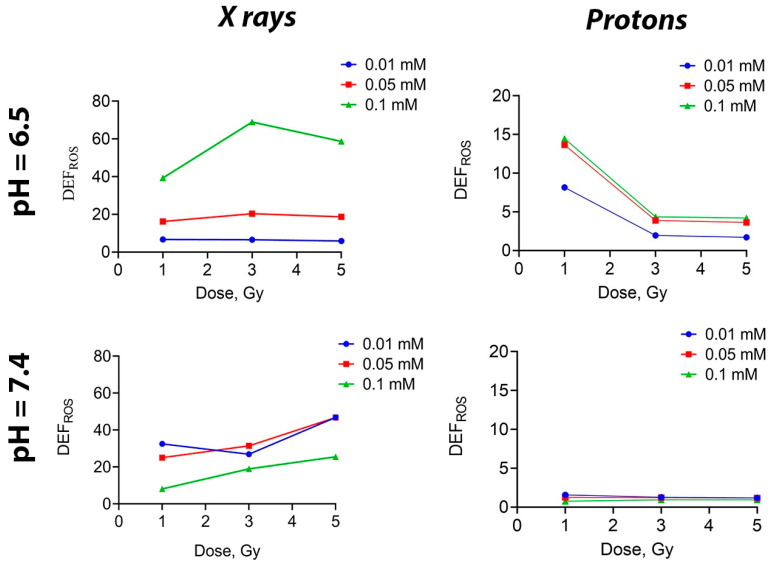
ROS generation by Gd_0.7_Ce_0.2_Tb_0.1_F_3_ NPs in solutions with slightly acidic (pH = 6.5) and physiological (pH = 7.4) pH under X-ray and proton beam irradiation at doses of 0–5 Gy.

To assess the potential of Gd_0.7_Ce_0.2_Tb_0.1_F_3_ NPs as a nanoradiosensitizer, we needed to confirm their ability to generate ROS when irradiated in solution. This would indicate their radiocatalytic activity. A fluorescent dye H2DCFDA was used to assess the total ROS level in solutions after exposure to ionizing radiation, according to the method described previously [54]. It was shown that when photon radiation was applied to a solution containing Gd_0.7_Ce_0.2_Tb_0.1_F_3_ NPs at concentrations of 0.05 and 0.01 mM, there was no difference in the level of ROS generation (regardless of pH) (Figure 3). On the contrary, at the highest concentration of 0.1 mM, there was a sharp increase in ROS level at a pH of 6.5. When solutions were irradiated with a proton beam, the ROS level decreased as the dose increased at pH 6.5, while at pH 7.4, it remained unchanged as the dose continued to increase, regardless of the Gd_0.7_Ce_0.2_Tb_0.1_F_3_ NPs concentration. Such a difference in ROS generation may be attributed to the aggregation of NPs at a higher concentration. Meanwhile, the lack of effects at high doses of irradiation can be explained by the saturation limit, meaning that all possible ROS have been formed in the final volume of liquid.

**Figure 4:**
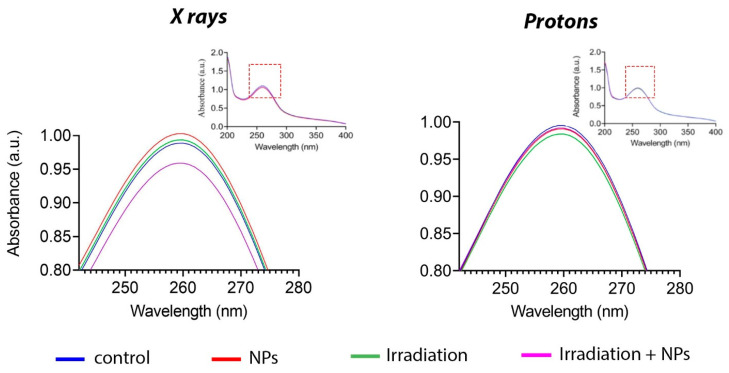
Superoxide anion radical (O_2_^•−^) generation by Gd_0.7_Ce_0.2_Tb_0.1_F_3_ NPs at a concentration of 5 mM under X-ray and proton beam irradiation.

One of the most highly reactive species formed during the radiolysis of water is superoxide anion radical (O_2_^•−^) [55]. To detect superoxide anion radicals, we used a specific dye—nitrotetrazolium blue. This dye is converted to formazan during reaction with the superoxide anion, which leads to a change in its absorption spectrum at 260 nm [56]. Figure 4 shows the change in the absorption spectrum following X-ray irradiation in the presence of Gd_0.7_Ce_0.2_Tb_0.1_F_3_ NPs at a concentration of 0.05 mM. At the same time, proton beam irradiation of a solution containing nanoparticles did not change the spectrum; it did not differ from the control group. Thus, it can be concluded that Gd_0.7_Ce_0.2_Tb_0.1_F_3_ NPs interact with photon radiation to generate superoxide anions, whereas their interaction with proton beam irradiation does not lead to the formation of reactive species.

**Figure 5:**
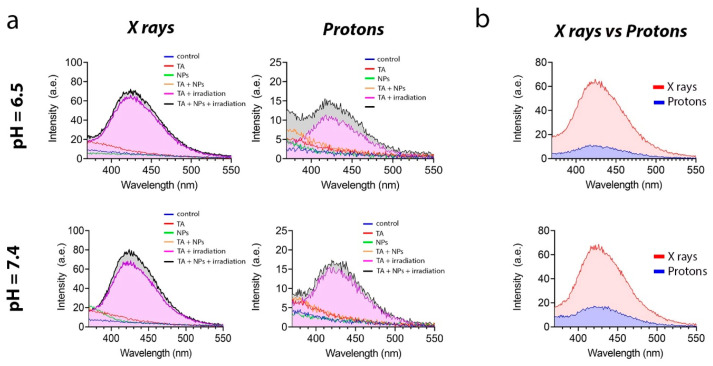
Hydroxyl radical (•OH) generation by Gd_0.7_Ce_0.2_Tb_0.1_F_3_ NPs at a concentration of 0.5 mM under X-ray and proton beam irradiation in solutions at slightly acidic (pH = 6.5) and physiological (pH = 7.4) conditions (**a**). Comparative analysis of X-ray irradiation with a proton beam irradiation for hydroxyl radical generation (**b**).

Next, we analyzed the ability of Gd_0.7_Ce_0.2_Tb_0.1_F_3_ NPs to generate hydroxyl radical (•OH) under irradiation (Figure 5). For this purpose, we used disodium terephthalate salt, which reacts with •OH, forming a fluorescent product, which can be detected spectrofluorometrically. Figure 5a shows that the fluorescence intensity at pH 6.5 was lower in both groups. This is presumably due to the fact that the medium is already saturated with free protons. These protons react with the hydroxyl radical more actively than the dye itself, which models the physiological features of radioresistant cancer cells to some extent. It should also be noted that the fluorescence intensity upon X-ray irradiation was much higher than upon proton beam irradiation (Figure 5b).

**Figure 6:**
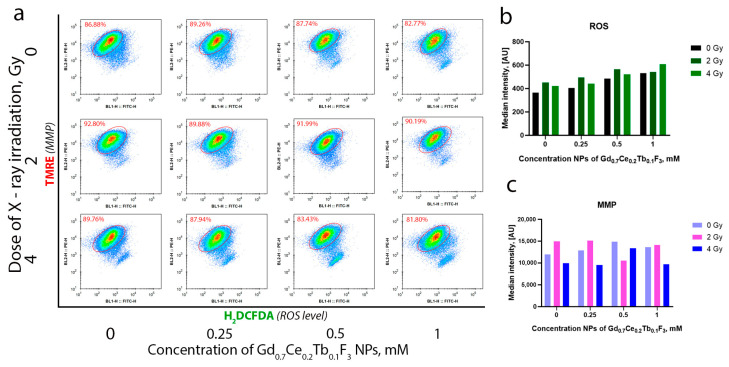
Combined effect of Gd_0.7_Ce_0.2_Tb_0.1_F_3_ NPs (0.25–1 mM) and X-ray irradiation (2 and 4 Gy) on the level of intracellular ROS (**b**) and MMP (**c**) in MCF-7 cells. Cells were gated at the E2 gate using a FITC/PE plot (**a**). The analysis was performed 3 h after irradiation.

Summarizing the results of the study on the radiation-induced redox activity of Gd_0.7_Ce_0.2_Tb_0.1_F_3_ NPs, we can confidently state that these NPs exhibit remarkable radiocatalytic activity under photon irradiation. This activity leads to the formation of various types of ROS at high levels during exposure. Consequently, to study the radiosensitizing effect of Gd_0.7_Ce_0.2_Tb_0.1_F_3_ NPs on MCF-7 cancer cells in vitro, we used X-ray irradiation. In the first stage, we analyzed the efficiency of cellular uptake of Gd_0.7_Ce_0.2_Tb_0.1_F_3_ NPs by flow cytometry based on side scatter intensity (SSC) analysis (Figure S6). This information is needed to confirm that the identified radiosensitization effects are associated with the activity of the nanoparticles. A concentration-dependent accumulation of Gd_0.7_Ce_0.2_Tb_0.1_F_3_ NPs was revealed, with the highest uptake observed at a concentration of 0.5 mM. This indicates that a concentration of 1 mM is excessive for this cell line. We propose that at a concentration of 1 mM, certain parts of nanoparticles are not directly located within cancer cells but are associated with the cell membrane. Studying the molecular mechanisms of radiation-induced cell damage, we analyzed the level of intracellular ROS and MMP by flow cytometry (Figure 6a). It was revealed that irradiation leads to an increase in the level of intracellular ROS, and the combined action of Gd_0.7_Ce_0.2_Tb_0.1_F_3_ NPs and X-ray enhances this effect (Figure 6b). The highest effect—more than a 2-fold increase relative to the unirradiated control—was observed with Gd_0.7_Ce_0.2_Tb_0.1_F_3_ NPs at a concentration of 1 mM under X-ray irradiation at a dose of 4 Gy. Moreover, a concentration- and dose-dependent MMP decrease was observed, indicating mitochondrial membrane depolarization under oxidative stress. This directly correlates with the obtained experimental data on the increase in the level of intracellular ROS after X-ray irradiation (Figure 6c). Thus, we conclude that Gd_0.7_Ce_0.2_Tb_0.1_F_3_ NPs are able to enter cancer cells and effectively accumulate within them. The NPs then trigger a cascade of radiocatalytic reactions and induce radiation-induced oxidative stress. Furthermore, the sharp increase in ROS levels correlates with data on the redox activity of Gd_0.7_Ce_0.2_Tb_0.1_F_3_ NPs obtained in acellular systems under X-ray irradiation, while the decrease in MMP likely reflects a disruption of homeostasis and redox status in the cancer cells.

**Figure 7:**
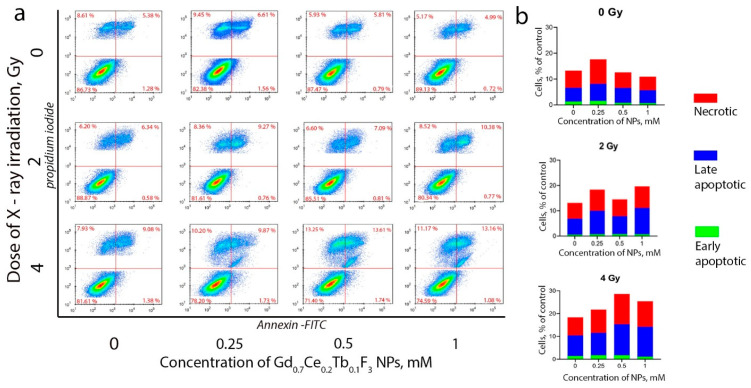
Combined effect of Gd_0.7_Ce_0.2_Tb_0.1_F_3_ NPs (0.25–1 mM) and X-ray irradiation (2 and 4 Gy) on cell death induction and distribution in MCF-7 cells (**b**). Cells were gated into living (FITC-/PE-), early apoptotic (FITC+/PE-), late apoptotic (FITC+/PE+), and necrotic (FITC-/PE+) cells (**a**). The analysis was performed 72 h after irradiation.

After evaluating the molecular mechanisms of the combined action of Gd_0.7_Ce_0.2_Tb_0.1_F_3_ NPs and photon irradiation, we analyzed cell death induction and distribution using flow cytometry (Figure 7). A pronounced radiation-induced sensitizing effect of Gd_0.7_Ce_0.2_Tb_0.1_F_3_ NPs at concentrations of 0.5 and 1 mM was demonstrated. The presented flow cytometry data accurately reflect the dynamics of the increase in cell death induction as the irradiation dose and concentration of NPs increase (Figure 7a). It was found that nanoparticles did not lead to an increase in the proportion of necrotic or apoptotic cells 72 h after exposure (Figure 7b). At the same time, the combined action of Gd_0.7_Ce_0.2_Tb_0.1_F_3_ NPs and X-ray irradiation at a dose of 2 Gy increased the proportion of late apoptotic and necrotic cells. An increase in the dose to 4 Gy resulted in a more remarkable radiosensitization effect, increasing the proportion of dead cells by more than two times compared to the non-irradiated control. It should be noted that the highest dose of 4 Gy led to the formation of an additional population of cells (Figure 7a), which is most likely due to the appearance of a large number of apoptotic bodies or parts of destroyed cells. Accordingly, we can clearly link the changes in cell death induction and distribution in the presence of Gd_0.7_Ce_0.2_Tb_0.1_F_3_ NPs under X-ray irradiation to the effects of radiosensitization, since the nanoparticles themselves do not increase the proportion of dead cells, and the effects are dose- and concentration-dependent.

In addition, we analyzed the combined action of Gd_0.7_Ce_0.2_Tb_0.1_F_3_ NPs and X-ray irradiation on the clonogenic ability of MCF-7 cells using a clonogenic assay (Figure S7). This analysis revealed a pronounced decrease in the number of colonies formed after the introduction of NPs (0.5 and 1 mM) and X-ray irradiation at a dose of 2 Gy. It should be noted that the Gd_0.7_Ce_0.2_Tb_0.1_F_3_ NPs themselves did not cause a decrease in the number of colonies at all of the concentrations studied (0–1 mM), while irradiation at a dose of 4 Gy led to complete inhibition of colony formation, even in the control group.

## 4. Discussion

The concept of creating an effective nanoradiosensitizer based on Gd_0.7_Ce_0.2_Tb_0.1_F_3_ NPs is to include several REE elements with different physicochemical properties necessary to impart both diagnostic and therapeutic modalities. Gadolinium is used not only as a heavy element that effectively absorbs ionizing radiation but also provides MRI contrast [57]. Ceria is a unique enzyme-mimicking element, which is able to effectively carry out catalytic reactions [58]. Terbium, with luminescent properties [59], perfectly enhances the effect of radioluminescence, which complements the exceptional radiocatalytic properties of Gd_0.7_Ce_0.2_Tb_0.1_F_3_ NPs. The aforementioned features of Gd_0.7_Ce_0.2_Tb_0.1_F_3_ NPs increase the efficiency of cancer cell killing under irradiation due to several mechanisms of action, which enhance each other. The radiosensitizing activity of Gd_0.7_Ce_0.2_Tb_0.1_F_3_ NPs, based on a multicomponent mechanism, may be more effective than the single-component composition proposed in the already approved first-in-class nanoradiosensitizer NBTRX3 [9,21,60,61].

The use of REE-based nanoparticles in radiosensitizers is dictated by their unique physicochemical properties. First of all, there is the high Z number and the large ionic radius of the atom due to the presence of the 4f shell, which contributes to a large shielding of the Coulomb potential. This mainly affects the ionization potential, which has the lowest value due to the large ion radius, leading to the formation of secondary photons and electrons. It is known that REE oxides have high catalytic activity due to defects in the crystal structure [62], and they also possess the properties of n-type semiconductors, which contribute to the effective photochemical degradation of substances [63]. In the case of REE fluoride-based nanomaterials, radioluminescence is the main mechanism of ROS generation. The presence of a fluoride ion reduces the phonon oscillation of the crystal lattice and promotes the efficient formation of excitons and secondary electrons, which effectively recombine in luminescent centers [64]. On the other hand, the basis for effective scintillation is photon upconversion, where at least two photons are sequentially absorbed and one high-energy photon is emitted. An important requirement for a nanoscintillator is the presence of a metastable absorbing state, which is necessary for the accumulation of photons. We assume that Gd_0.7_Ce_0.2_Tb_0.1_F_3_ NPs act according to the energy transfer mechanism due to the presence of Tb ions, which allows the re-emission of multiple low-energy photons in the UV range, thereby contributing to the generation of ROS [65]. Jacobsohn et al. demonstrated that doping of fluoride nanoparticles with REE ions effectively allows photons to be captured in a wide frequency range, thereby increasing scintillation [66]. However, the effect we have demonstrated requires further study, which will allow us to evaluate the radioluminescent properties of Gd_0.7_Ce_0.2_Tb_0.1_F_3_ NPs after exposure to protons and X-rays.

It is worth noting that the cytotoxicity of nanomaterials strongly depends on the shape, size, and charge of the surface [67,68,69,70,71]. The synthesized Gd_0.7_Ce_0.2_Tb_0.1_F_3_ NPs have a spherical shape and citrate as a biocompatible stabilizer, which provides the necessary level of biocompatibility. At the same time, the cytotoxic effects of nanoparticles also strongly depend on the efficiency of accumulation in each cell type and their intracellular localization and processing (for example, partial dissolution and the appearance of metal ions) [72]. We analyzed the cytotoxic effects of Gd_0.7_Ce_0.2_Tb_0.1_F_3_ NPs on normal and tumor cells, revealing IC50 values, which allowed us to assess the possible contribution of toxicity in the analysis of radiosensitizing properties, and also did not reveal any toxic effects during intraperitoneal administration of nanoparticles in vivo. This indicates the prospects of using such nanoparticles as an effective radiosensitizer.

## 5. Conclusions

In the present study, we synthesized and comprehensively characterized Gd_0.7_Ce_0.2_Tb_0.1_F_3_ NPs. Studies of the radiocatalytic activity of Gd_0.7_Ce_0.2_Tb_0.1_F_3_ NPs in aqueous solutions demonstrated a more pronounced pH-dependent effect of ROS generation precisely when irradiated with photons rather than protons. In this regard, we further investigated the molecular mechanisms of their activity in a culture of MCF-7 tumor cells exposed to X-ray irradiation in vitro. The molecular mechanisms of radiosensitization of Gd_0.7_Ce_0.2_Tb_0.1_F_3_ NPs under photon radiation are due to their ability to effectively generate ROS, which in turn cause significant damage to cellular structures, an increase in intracellular ROS levels, and disruption of cellular metabolism, leading to the death of tumor cells. Multifunctional Gd_0.7_Ce_0.2_Tb_0.1_F_3_ NPs have great potential for improving photon radiotherapy, considering their high radiosensitizing effects through pronounced radiocatalytic activity.

## Data Availability

Data is contained within the article or Supplementary Materials.

## Supplementary Materials

The following supporting information can be downloaded at: https://www.mdpi.com/article/10.3390/biomedicines13071537/s1, Figure S1: Complete scheme of experiments with Gd_0.7_Ce_0.2_Tb_0.1_F_3_ NPs; Figure S2: Size distribution of Gd_0.7_Ce_0.2_Tb_0.1_F_3_ NPs in different buffers (MQ, DMEM/F12, DMEM/F12+10% FBS); Figure S3: Micrographs of cells 72 h after coincubation with Gd_0.7_Ce_0.2_Tb_0.1_F_3_ NPs. Fluorescent labels with propidium iodide (dead cells)/Hoechst 33,342 (all cells). The scale bar is 10 µm; Figure S4: Micrographs of cells 72 h after coincubation with Gd_0.7_Ce_0.2_Tb_0.1_F_3_ NPs. Fluorescent labels with TMRE (mitochondrial membrane potential). The scale bar is 10 µm; Figure S5: Analysis of animal survival after intraperitoneal injection of nanoparticles for 20 days. Control animals received an injection of saline solution (0.9% NaCl). The solutions were administered intraperitoneally at a volume of 0.3 mL per mouse; Figure S6: Uptake of Gd_0.7_Ce_0.2_Tb_0.1_F_3_ NPs by MCF-7 cells after 16 h of coincubation. Granularity represents side scatter intensity; Figure S7: Combined effect of Gd_0.7_Ce_0.2_Tb_0.1_F_3_ NPs (0.25–1 mM) and X-ray irradiation (2 and 4 Gy) on the clonogenic activity of MCF-7 cells. Results are presented as mean ± SD. Table S1: Colloidal stability of Gd_0.7_Ce_0.2_Tb_0.1_F_3_ NPs after 28 days of storage.
