# Supplementary material for: Novel Cerium- and Terbium-Doped Gadolinium Fluoride Nanoparticles as Radiosensitizers with Pronounced Radiocatalytic Activity
Source: Biomedicines. 2025 Jun 24;13(7):1537. doi: 10.3390/biomedicines13071537 (PMC12293020; doi:10.3390/biomedicines13071537)
Supplement: Supplementary file 1 [file biomedicines-13-01537-s001.zip › biomedicines-3645414-supplementary.pdf]

Supplement materials

Novel Cerium and Terbium Doped Gadolinium Fluoride Nanoparticles as Radiosensitizer with Pronounced Radiocatalytic Activity

Nikita A. Pivovarov, Danil D. Kolmanovich, Nikita N. Chukavin, Irina V. Savintseva, Nelli R. Popova, Alexander E. Shemyakov, Arina D. Filippova, Maria A. Teplonogova, Alexandra V. Yurkovskaya, Ivan. V. Zhukov, Azamat Y. Akkizov, Anton L. Popov

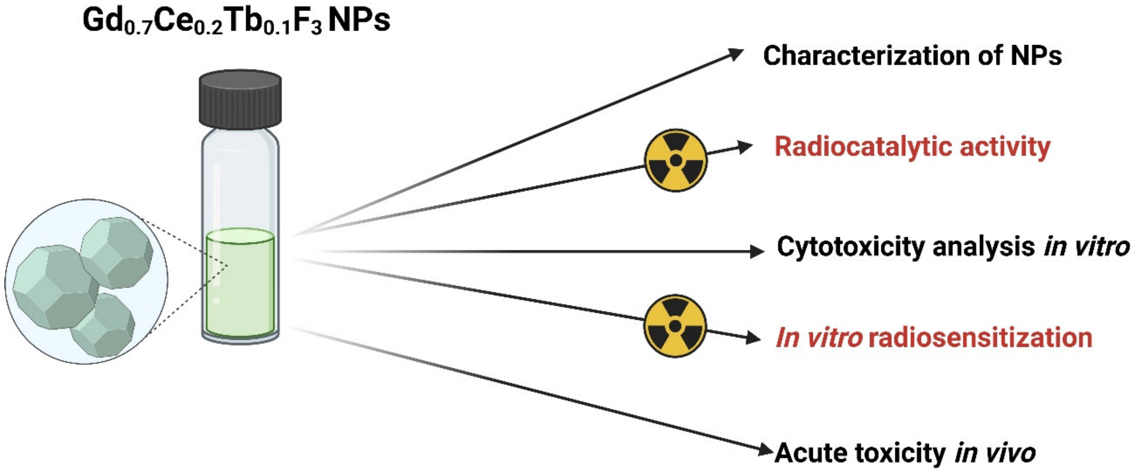

Figure S1. The complete scheme of experiments with $Gd_{0.7}Ce_{0.2}Tb_{0.1}F_3$ NPs.

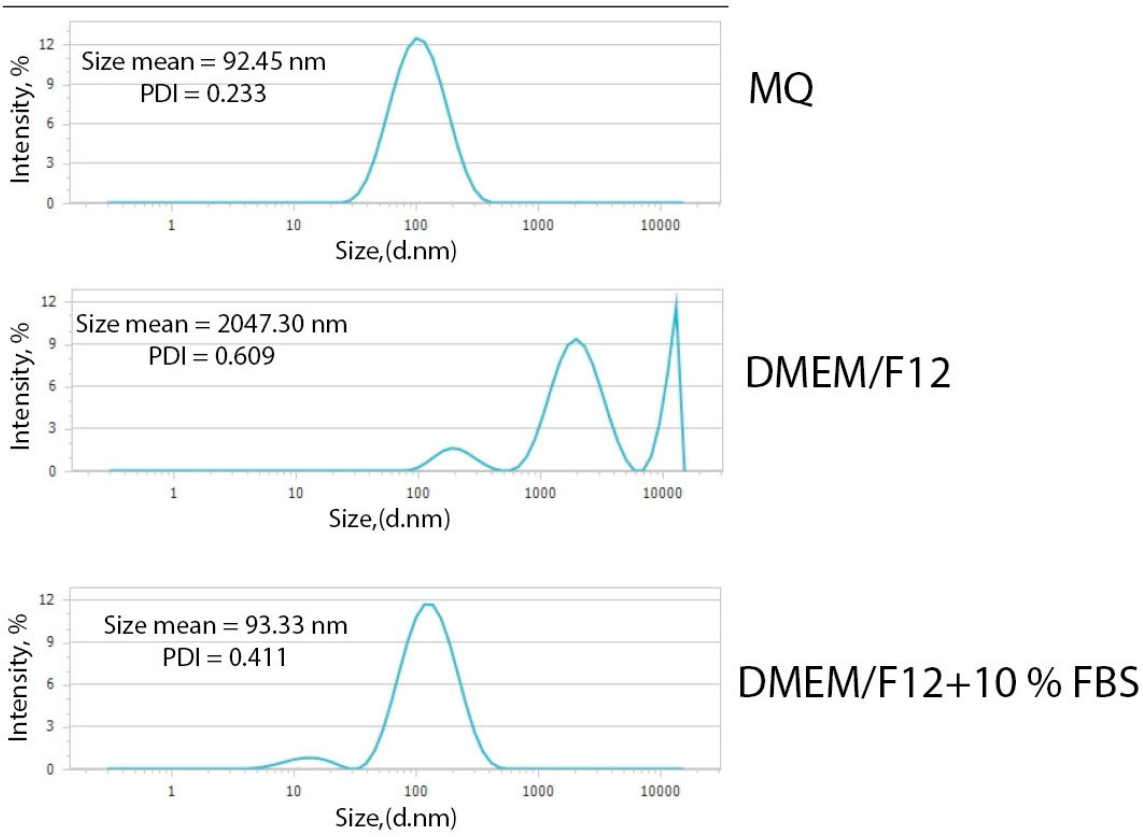

Figure S2. Size distribution of $Gd_{0.7}Ce_{0.2}Tb_{0.1}F_3$ NPs in different buffers (MQ, DMEM/F12, DMEM/F12+10% FBS).

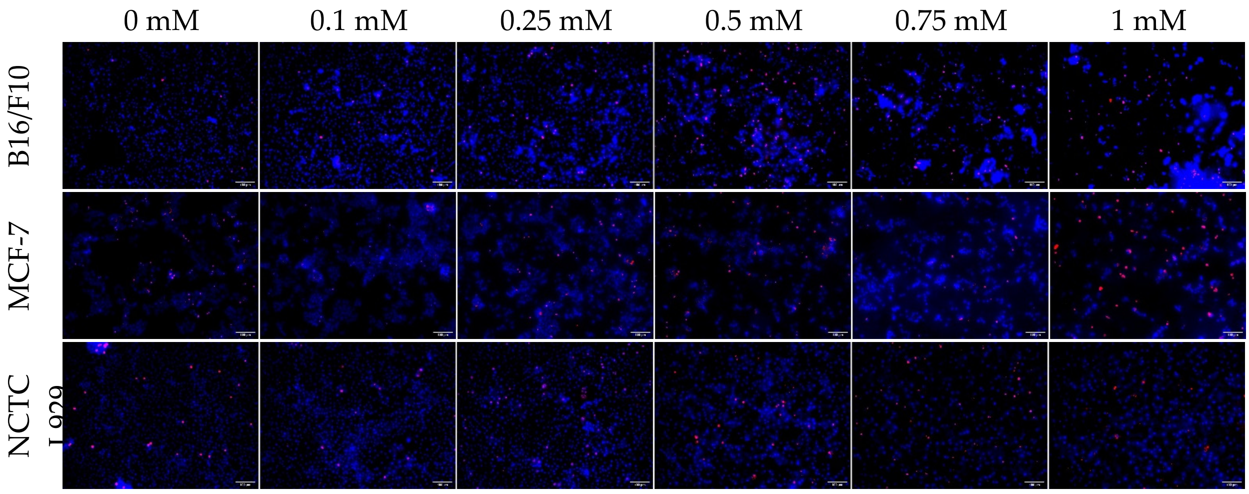

Figure S3. Micrographs of cells 72 hours after coincubation with $Gd_{0.7}Ce_{0.2}Tb_{0.1}F_3$ NPs. Fluorescent labels with propidium iodide (dead cells)/Hoechst 33342 (all cells). The scale bar is 10 μm.

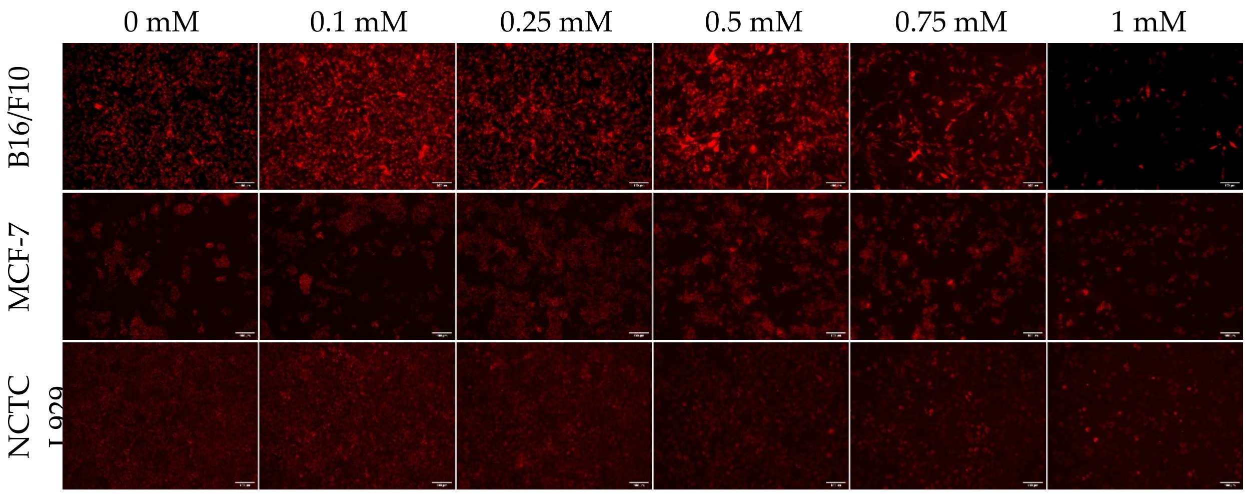

Figure S4. Micrographs of cells 72 hours after coincubation with $Gd_{0.7}Ce_{0.2}Tb_{0.1}F_3$ NPs. Fluorescent labels with TMRE (mitochondrial membrane potential). The scale bar is 10 μm.

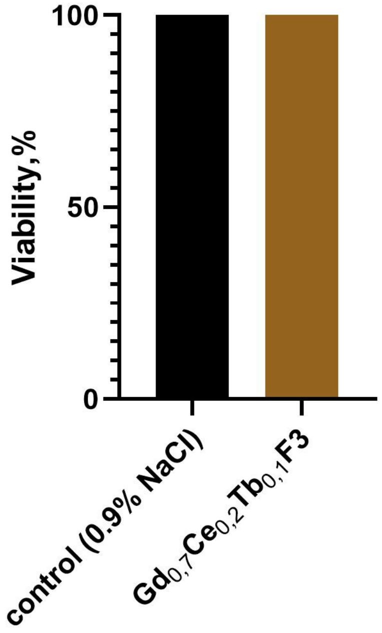

Figure S5. Analysis of animal survival after intraperitoneal injection of nanoparticles for 20 days. Control animals received a saline solution (0.9 % NaCl) for injection. The solutions were administered intraperitoneally in a volume of 0.3 mL per mouse.

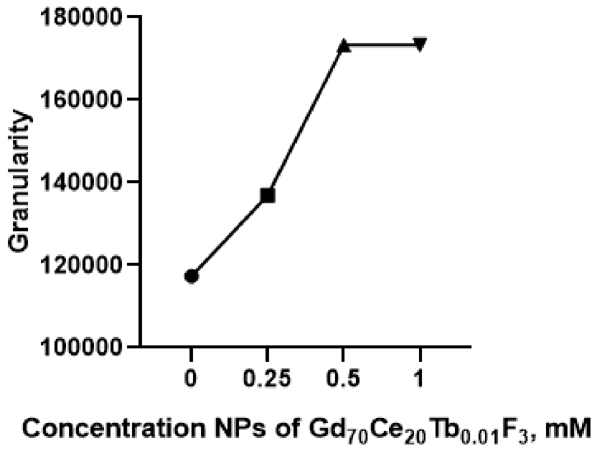

Figure S6. Uptake of $Gd_{0.7}Ce_{0.2}Tb_{0.1}F_3$ NPs by MCF-7 cells after 16 hours of coincubation. The granularity represents side scatter intensity value.

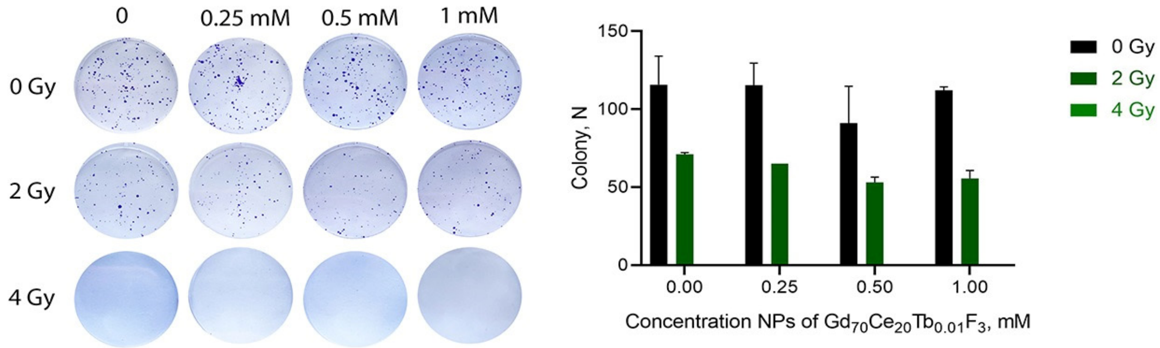

Figure S7. Combined effect of $Gd_{0.7}Ce_{0.2}Tb_{0.1}F_3$ NPs (0.25-1 mM) and X-ray irradiation (2 and 4 Gy) on the clonogenic activity of MCF-7 cells. Results are presented as mean ± SD.

Table S1. Colloidal stability of $Gd_{0.7}Ce_{0.2}Tb_{0.1}F_3$ NPs after 28 days of storage

Size (nm) in H_2O (18.2 MOm·sm)				
**0 day**	**7 days**	**14 days**	**21 days**	**28 days**
112,57	126.63	123.05	130.61	122.17
121,93	124.09	125.15	124.56	119.92
122,8	124.85	123.68	119.39	119.04
Zeta (mV) in H_2O (pH=7.2)				
**0 day**	**7 days**	**14 days**	**21 days**	**28 days**
-17.39	-17.08	-13.60	-17.32	-17.11
-17.00	-21.01	-17.09	-15.59	-12.73
-17.81	-16.11	-18.14	-15.11	-15.17
